# HIV care using differentiated service delivery during the COVID‐19 pandemic: a nationwide cohort study in the US Department of Veterans Affairs

**DOI:** 10.1002/jia2.25810

**Published:** 2021-10-28

**Authors:** Kathleen A. McGinnis, Melissa Skanderson, Amy C. Justice, Kathleen M. Akgün, Janet P. Tate, Joseph T. King, Christopher T. Rentsch, Vincent C. Marconi, Evelyn Hsieh, Christopher Ruser, Farah Kidwai‐Khan, Roozbeh Yousefzadeh, Joseph Erdos, Lesley S. Park

**Affiliations:** ^1^ VA CT Healthcare System US Department of Veterans Affairs West Haven Connecticut USA; ^2^ Internal Medicine Yale School of Medicine New Haven Connecticut USA; ^3^ Epidemiology and Population Health London School of Hygiene and Tropical Medicine London UK; ^4^ Emory University School of Medicine, Rollins School of Public Health and the Atlanta VA Medical Center Atlanta Georgia USA; ^5^ Stanford Center for Population Health Sciences, Department of Epidemiology and Population Health Stanford School of Medicine Stanford California USA

**Keywords:** COVID‐19, extended refills, HIV, veterans, virtual visits

## Abstract

**Introduction:**

The Department of Veterans Affairs (VA) is the largest provider of HIV care in the United States. Changes in healthcare delivery became necessary with the COVID‐19 pandemic. We compared HIV healthcare delivery during the first year of the COVID‐19 pandemic to a prior similar calendar period.

**Methods:**

We included 27,674 people with HIV (PWH) enrolled in the Veterans Aging Cohort Study prior to 1 March 2019, with ≥1 healthcare encounter from 1 March 2019 to 29 February 2020 (2019) and/or 1 March 2020 to 28 February 2021 (2020). We counted monthly general medicine/infectious disease (GM/ID) clinic visits and HIV‐1 RNA viral load (VL) tests. We determined the percentage with ≥1 clinic visit (in‐person vs. telephone/video [virtual]) and ≥1 VL test (detectable vs. suppressed) for 2019 and 2020. Using pharmacy records, we summarized antiretroviral (ARV) medication refill length (<90 vs. ≥90 days) and monthly ARV coverage.

**Results:**

Most patients had ≥1 GM/ID visit in 2019 (96%) and 2020 (95%). For 2019, 27% of visits were virtual compared to 64% in 2020. In 2019, 82% had VL measured compared to 74% in 2020. Of those with VL measured, 92% and 91% had suppressed VL in 2019 and 2020. ARV refills for ≥90 days increased from 39% in 2019 to 51% in 2020. ARV coverage was similar for all months of 2019 and 2020 ranging from 76% to 80% except for March 2019 (72%). Women were less likely than men to be on ARVs or to have a VL test in both years.

**Conclusions:**

During the COVID‐19 pandemic, the VA increased the use of virtual visits and longer ARV refills, while maintaining a high percentage of patients with suppressed VL among those with VL measured. Despite decreased in‐person services during the pandemic, access to ARVs was not disrupted. More follow‐up time is needed to determine whether overall health was impacted by the use of differentiated service delivery and to evaluate whether a long‐term shift to increased virtual healthcare could be beneficial, particularly for PWH in rural areas or with transportation barriers. Programmes to increase ARV use and VL testing for women are needed.

## 1 INTRODUCTION

The United States (US) Department of Veterans Affairs (VA) provides healthcare at no or low cost to eligible veterans and is delivered mainly through VA clinics and facilities. The VA benefits from one of the most highly developed health information systems in the world [1] and is one of the first US healthcare systems to create extensive virtual healthcare infrastructure including telephone and video visits (henceforth referred to as “virtual”) [2, 3]. With the emergence of the COVID‐19 pandemic, changes in healthcare delivery became immediately necessary [4, 5], and the VA responded quickly by expanding virtual care [2, 6, 7, 8, 9, 10]. On 19 March 2020, the VA issued guidance for “Alternative Telehealth Communication Technologies During COVID19 National Emergency” [11]. This guidance outlined the preferred modes of communication including VA Video Connect and government‐furnished phones, and also allowed for the use of alternative technologies to “augment clinical activities related to providing care to patients” during the COVID‐19 pandemic [11]. By June 2020, 58% of VA visits were virtual compared to 14% prior to March 2020 [12].

The VA is the largest provider of HIV care in the United States [1, 13]. People with HIV (PWH) need consistent HIV healthcare engagement to maintain antiretroviral (ARV) medication adherence and HIV‐1 plasma RNA viral load (VL) suppression [14, 15, 16]. Substance use screening is also an important component of HIV care [17]. Since 2015, the World Health Organization has promoted “differentiated service delivery” for PWH to simplify access to care and to reduce time spent in healthcare facilities [18]. One study reported that 5% of PWH at three VA sites used telehealth during the 3–4 years prior to the COVID‐19 pandemic [19]. The pre‐pandemic criteria for virtual visits prioritized patients who were virologically suppressed but had transportation or clinic distance challenges.

During in‐person visits, patients are typically screened by support staff annually about health related items including alcohol and tobacco use [20] prior to seeing the clinician. In contrast, during virtual visits clinicians are expected to administer these screenings in addition to adjusting to the use of technology and any corresponding trouble‐shooting issues for themselves or patients [2]. For those who utilize alcohol and tobacco use data collected via screenings, this transition to increased use of virtual visits during the COVID‐19 pandemic could potentially lead to gaps in information and care.

Among women receiving HIV care within the VA, a lower percentage had ARV coverage and suppressed VL compared to men with HIV [21], and some studies have identified an association between race/ethnicity and lower virtual healthcare use [22]. Whether the impact of the COVID‐19 pandemic on HIV healthcare varies by race/ethnicity and/or gender is unknown.

Understanding how the pandemic has impacted HIV healthcare service utilization, ARV adherence and substance use disorder screening can inform efforts to maintain continuity of care for PWH and other chronic health conditions using differentiated service delivery. More specifically, examining the impact of healthcare changes for PWH may provide broader insights into the implications of virtual care models for other chronic diseases as well as for maximizing healthcare resources and/or helping overcome barriers to care such as distance to clinic and/or lack of mobility or transportation [5]. Our main goals were to compare among PWH during and prior to the COVID‐19 pandemic: (1) HIV healthcare delivery and (2) frequency of alcohol and tobacco use screening. Secondary aims included (1) comparing HIV healthcare delivery by race/ethnicity and gender and (2) evaluating diagnoses for alcohol use disorder (AUD) and tobacco use/smoking during and prior to the pandemic.

## 2 METHODS

### 2.1 Data source – Veterans Aging Cohort Study

The Veterans Aging Cohort Study (VACS) is a national cohort of 60,055 PWH and 125,122 age‐matched, race/ethnicity‐matched, sex‐matched and clinical site‐matched people without HIV who were identified in the US VA electronic health record in the fiscal years 1997–2020 using a modified existing algorithm [1]. Data were extracted from the VA Corporate Data Warehouse, a national repository that incorporates data from clinical and administrative systems into a data warehouse structure [23]. This study was approved by the Institutional Review Boards of the VA Connecticut Healthcare System and Yale University School of Medicine and has been granted a waiver of informed consent.

Within VACS, we identified PWH who entered care prior to 1 March 2019 with evidence of at least one outpatient VA healthcare encounter of any type including, but not limited to, general medicine (GM), infectious disease (ID), emergency care, mental health, pharmacy and laboratory from 1 March 2019 to 28 February 2021, and were alive at the end of the study period (28 February 2021). Due to the emerging body of evidence regarding health needs for persons infected with SARS‐CoV‐2, we excluded 1524 PWH who had an indication of a positive SARS‐CoV‐2 PCR laboratory test up to 28 February 2021. The analytic sample included 27,674 PWH.

### 2.2 Variable definitions

Age, race/ethnicity and sex were determined as of 1 March 2019. We identified GM/ID clinic visits related to HIV primary care (VA clinic stop codes: 27, 170, 172, 301, 310, 318, 319, 323, 324, 338, 348, 349, 350, 311). Virtual (video or telephone) visits were determined based on methodology adapted from Ferguson and colleagues [12]. HIV‐1 RNA VL was categorized as suppressed (≤50 copies/ml), detectable (>50 copies/ml) and not measured. ARV pharmacy fill/refill length was categorized as <60, 60–89 and ≥90 days.

We identified PWH who were screened with the Alcohol Use Disorder Identification Test‐Consumption (AUDIT‐C) and for tobacco use history via the clinical reminder system [19, 20]. Unhealthy alcohol use was based on AUDIT‐C ≥ 4 for men or ≥3 for women [24]. Tobacco use was identified as current, past or never based on responses to two clinical reminder questions: “Do you smoke cigarettes or use tobacco every day, some days, or not at all”?; and those who responded “not at all” were asked about former or never use. For those with multiple responses per year, we used the response representing the highest level of use. Additionally, we identified for each year those with at least one outpatient International Classification of Diseases, Tenth Revision, Clinical Modification (ICD‐10) diagnosis code for AUD using codes F10.1x, F10.2x [25] or tobacco use/smoking using codes Z72.0x, F17.21, Z87.891.

### 2.3 Analyses

For each month of 1 March 2019 to 29 February 2020 (2019) and 1 March 2020 to 28 February 2021 (2020), we summarized counts of GM/ID clinic visits (in‐person vs. virtual), HIV‐1 RNA VL tests, ARV prescriptions by length, AUDIT‐C administered and tobacco use responses collected. We also calculated the percentage of patients with ARV coverage for each month based on prescription fill dates, days, supply and an additional half day supply was added to approximate lag time and overlap of fills.

For each period in 2019 and 2020 we calculated the percentage of individuals with ≥1 clinical encounter (by type), ≥1 HIV‐1 RNA VL test (by detectable/suppressed) and any ARV use. We determined whether having ≥1 clinical encounter, ≥1 HIV‐1 RNA VL test, or any ARV use varied by race/ethnicity and gender. Additionally, we calculated the percentage screened for AUDIT‐C and tobacco use along with corresponding responses for each year. Lastly, we calculated the percentage with an ICD‐10 diagnosis for alcohol use disorder (AUD) or smoking/tobacco use for each year.

## 3 RESULTS

**Figure 1:**
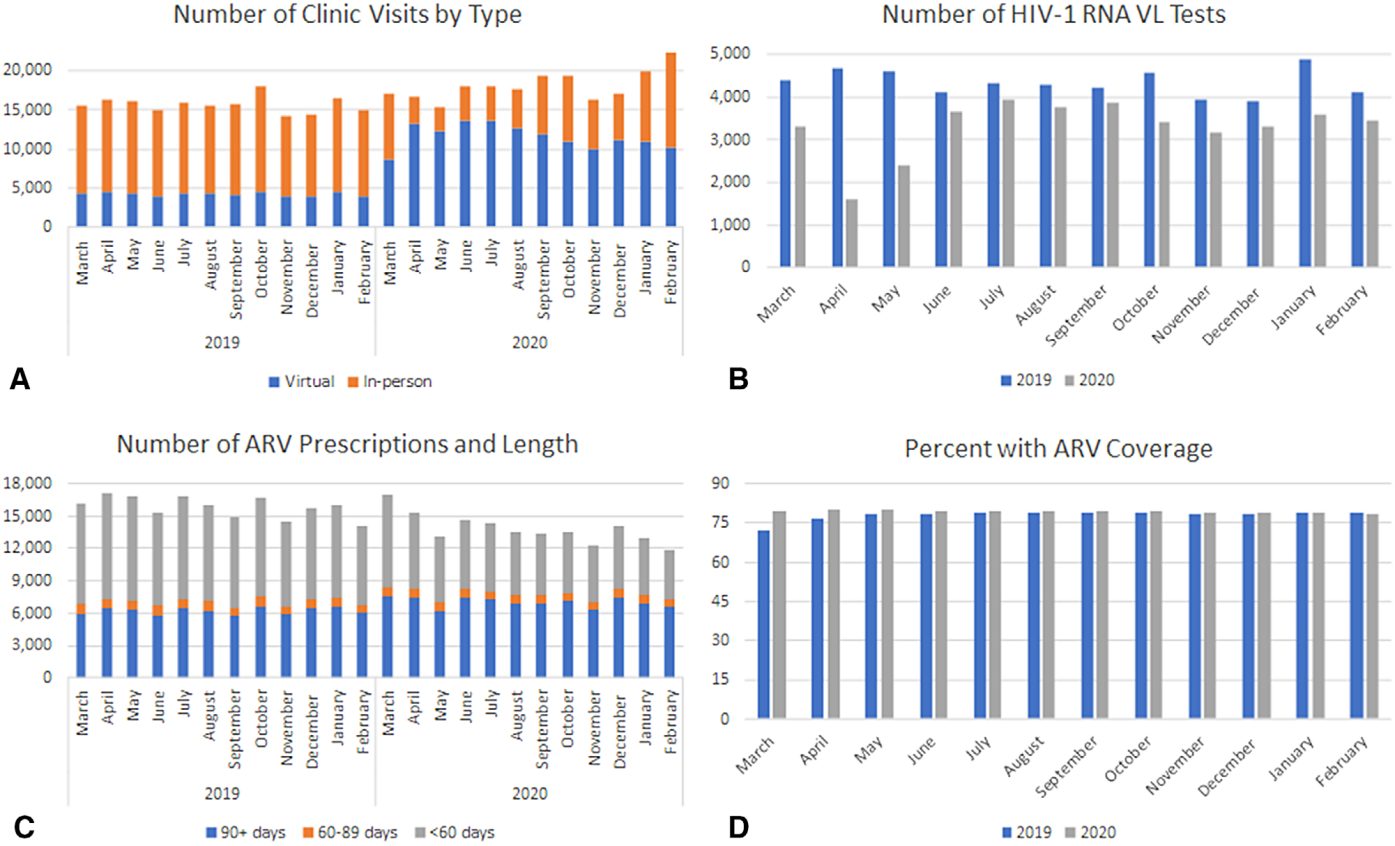
Healthcare services among 27,674 PWH in 2019 and 2020 ARV, antiretroviral therapy; VL, viral load; 2019, 1 March 2019 to 28 February 2020; 2020, 1 March 2020 to 29 February 2021.

Of the 27,674 PWH enrolled in VACS prior to March 2019 and with at least one healthcare encounter in 2019 or 2020, the median age was 59 years (range = 23–97), 96% were men, 45% non‐Hispanic Black (Black), 35% non‐Hispanic White (White), 8% Hispanic, 3% other (American Indian, Asian, Pacific Islander or mixed race) and 9% unknown (Table 1). Almost everyone had at least one healthcare encounter of any type in 2019 (99%; 27,493) and 2020 (98%; 27,107). For all months except May, there were more visits in 2020 than in 2019 (Figure 1a). The transition to a higher percentage of virtual visits started in March 2020 and continued throughout February 2021 (Figure 1a). In 2019, 27% of visits were virtual compared to 64% in 2020. Of the virtual visits, 99% and 92% were by telephone (vs. video) in 2019 and 2020, respectively. Considering GM/ID clinics specifically, almost all patients had at least one virtual or in‐person GM/ID clinic visit in 2019 (96%) and 2020 (95%) and this was similar for those of Black, White and Hispanic race/ethnicity and for men and women. For those of other or unknown race/ethnicity, the percentage with at least one GM/ID encounter in 2020 was slightly lower – 93% and 91%, respectively (Table 2). Compared to men, women were less likely to have in‐person visits (91% vs. 95% in 2019 and 77% vs. 80% in 2020) and more likely to have virtual visits (62% vs. 57% in 2019 and 92% vs. 89% in 2020). Having any GM/ID clinic visits was similar by gender in both years ranging from 94% to 96% (Table 2).

**Table 1:** Characteristics of people with HIV in the Veterans Aging Cohort Study (*n* = 27,674)

Characteristics		
Mean age in years (SD)	59 (12.1)	
Race/ethnicity (%)		
Black	45	
White	35	
Hispanic	8	
Other	3	
Unknown	9	
Gender (%)		
Men	96	
Women	4	
	Year	
HIV healthcare (%)	2019	2020
GM/ID visit		
Any	96	95
In person	95	80
Virtual	57	89
HIV‐1 RNA VL test detectable	6	7
HIV‐1 RNA VL test suppressed	76	67
No test	18	26
On ARVS	85	84
Alcohol use (%)		
AUDIT‐C – clinical reminder		
0 (no use)	35	29
1–3/1–4 (some use)	30	23
>3/>4 (unhealthy use)	10	7
Not asked	25	40
Alcohol use disorder diagnosis	11	9
Tobacco use/smoking (%)		
Clinical reminder		
Current	27	20
Past	23	19
Never	24	21
Not asked	26	40
Diagnosis	20	18

Abbreviations: ARV, antiretroviral therapy; AUDIT‐C, Alcohol use disorder identification test‐consumption; GM/ID, general medicine/infectious diseases; SD, standard deviation; VL, HIV‐1 RNA viral load; 2019, 1 March 2019 to 28 February 2020; 2020, 1 March 2020 to 29 February 2021.

**Table 2:** HIV healthcare by race/ethnicity and gender in 2019 and 2020 (*n* = 27,674)

		GM/ID visit						VL		On	
		Any (%)		In person (%)		Virtual (%)		Measured (%)		ARVs (%)	
Race/ethnicity	*N*	2019	2020	2019	2020	2019	2020	2019	2020	2019	2020
Black	12,577	96	95	96	81	56	90	83	75	85	85
White	9759	96	95	96	81	59	90	82	75	86	85
Hispanic	2167	96	95	95	82	60	91	86	78	86	86
Other	713	96	93	94	78	60	87	83	73	86	85
Unknown	2485	93	91	91	71	53	84	70	63	75	75
Gender											
Men	26,661	96	95	95	80	57	89	82	74	85	85
Women	1013	94	95	91	77	62	92	73	67	75	74

Abbreviations: ARV, antiretroviral therapy; GM/ID, general medicine/infectious diseases; VL, HIV‐1 RNA viral load; 2019, 1 March 2019 to 28 February 2020; 2020, 1 March 2020 to 29 February 2021.

There were fewer HIV‐1 RNA VL tests in 2020, particularly in the months of April and May (Figure 1b). In 2019, 82% had VL measured compared to 74% in 2020. Of those with VL measured, 92% and 91% had suppressed VL in 2019 and 2020, respectively. The percentage of PWH with VL measured in 2019 was similar among those of Black, White and other race/ethnicity (82%–83%), and slightly higher among Hispanic PWH (86%) and lower among those with unknown race/ethnicity (70%). The pattern was similar for 2020; the percentage with VL in 2020 was similar among those of Black, White and other race/ethnicity (73%–75%), and slightly higher among Hispanic PWH (78%) and lower among those with unknown race/ethnicity (63%) (Table 2). Compared to men, women were less likely to have VL measured in 2019 (73% vs. 82%) and 2020 (67% vs. 74%) (Table 2).

There were fewer ARV refills in all months of 2020 compared to 2019 except for in March. However, the percentage with a refill of ≥90 days was higher for all months in 2020 (Figure 1c). Overall, in 2020 51% of refills were for ≥90 days compared to 39% in 2019. Even though there was a lower number of refills in 2020 compared to 2019, because average prescription length was longer, ARV coverage was similar for all months of 2019 and 2020, ranging from 76% to 80% for all months except for March 2019 (72%) (Figure 1d). In 2019 and 2020, 85% and 84% had any ARV use, respectively. The percentage with ARV coverage was similar for those of Black, White, Hispanic and other race/ethnicity for both 2019 and 2020 (ranging from 85% to 86%). For those of unknown race/ethnicity, only 75% had any ARV use in both years (Table 2). Women were less likely than men to have ARV coverage in 2019 (75% vs. 85%) and 2020 (74% vs. 85%). There was little change in ARV coverage for either gender during the study period (Table 2).

**Figure 2:**
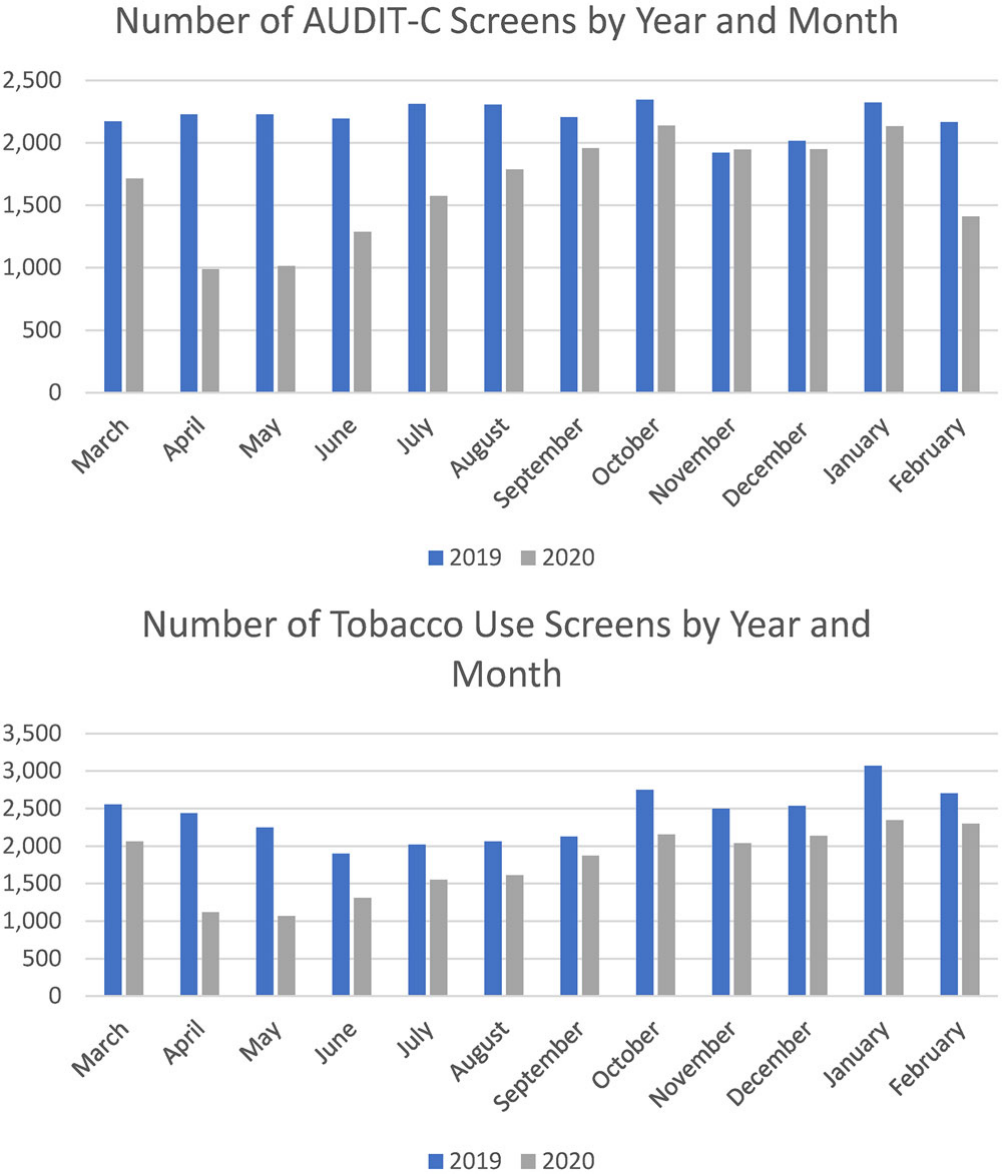
Screening for alcohol and tobacco use among 27,674 PWH in 2019 and 2020 AUDIT‐C, Alcohol Use Disorder Identification Test‐Consumption; 2019, 1 March 2019 to 28 February 2020; 2020, 1 March 2020 to 29 February 2021.

AUDIT‐C was collected less frequently for all months in 2020 compared to 2019, but only slightly less frequently in November and December (Figure 2). The AUDIT‐C was completed for 75% of PWH in 2019 and 60% in 2020 (Table 1). In 2019, 10% had an AUDIT‐C score indicating unhealthy alcohol use compared to 7% in 2020. In 2019, of those who only had a virtual visit, 25% had AUDIT‐C responses compared to 73% of those with only an in‐person visit. However, in 2020 the percentage with AUDIT‐C responses was similar for those with only virtual compared to only in‐person visits (44% vs. 46%). In 2019, 10% had an AUD diagnosis compared to 8% in 2020.

Tobacco use was collected less frequently for all months in 2020 compared to 2019 (Figure 2). The tobacco use items collected through the clinical reminder system were completed for 74% in 2019 and 60% in 2020. In 2019, 27% reported current tobacco use and 23% past tobacco use; in 2020, 20% reported current tobacco use and 19% reported past tobacco use (Table 1). In 2019, of those with only virtual visits, 21% had any tobacco use responses compared to 72% of those with only an in‐person visit. However, in 2020 the percentage with tobacco use information was more similar for those with only virtual compared to only in‐person visits (46% vs. 44%). In 2019, 17% had a tobacco use diagnosis compared to 15% in 2020.

## 4 DISCUSSION

In response to the COVID‐19 pandemic, the US VA ramped up the use of virtual visits (over 90% of virtual visits were telephone based) and increased refill length for ARVs for PWH. Despite a lower level of in‐person care and HIV‐1 RNA VL tests during the pandemic, the percentage with suppressed HIV‐1 RNA VL remained similar (among those for whom it was measured) and access to ARVs was maintained. HIV healthcare before and during the pandemic was similar for those of Black, White, Hispanic and other race/ethnicities and for men and women. However, alcohol and tobacco use screening occurred less frequently among PWH with the increased use of virtual care during the pandemic.

The finding of increased virtual visits for PWH from 27% in 2019 to 64% in 2020 is consistent with findings of other (non‐HIV specific) studies earlier in the pandemic [2, 12] as well as with VA guidance and support in the form of additional training and tablets that were provided for virtual visits. Ferguson et al. reported that virtual visits increased from 14% prior to COVID‐19 to 58% in June 2020; they also noted that virtual visits were more common among those with higher clinical or social needs [12]. During the pandemic, the VA Central Office recommended that newly diagnosed PWH with an opportunistic infection, low CD4 count, or serious ARV adverse event should be seen in an expedited matter in‐person or virtually, depending on patient preference. In‐person visits in ID clinics were restricted to patients with urgent care needs without COVID‐19 symptoms, patients presenting for same day ARV and routinely scheduled patients who were considered high risk (e.g., active opportunistic infections, high VL and low CD4 count).

The number of VL tests was particularly low in April and May 2020 (early in the pandemic). While routine VA laboratory testing was available throughout the pandemic, but with a transition from mostly “walk‐in” phlebotomy in close quarters to socially distanced appointments and limited to provider‐defined essential blood draws early in the pandemic.

While the number of ARV prescriptions was lower in 2020 compared to 2019, the percentage of prescriptions over 90 days was greater in 2020 compared to 2019. ARV prescriptions were refilled automatically (mostly via mail service) regardless of prior appointment attendance (encouraged but not mandatory) or whether laboratory testing was done. The VA has one of the most highly rated prescription mail order services that was providing around 80% of VA outpatient medications even before the start of the COVID‐19 pandemic [26, 27]. This differentiated service delivery likely contributed to ARV coverage being maintained throughout the pandemic.

Several studies have reported that those of Black and Hispanic race/ethnicity have been disproportionately negatively impacted by COVID‐19 with regard to testing, positivity rates and the vaccine rollout [28, 29, 30]. In this study of PWH receiving care in the VA, we found that HIV care during and prior to the COVID‐19 pandemic was similar by race/ethnicity except for those of unknown race/ethnicity and this is consistent with a previous study that reported that HIV clinical management and adherence in the VA was similar by race/ethnicity [31]. Because having unknown race/ethnicity in the VA is also associated with having fewer VA visits, this finding is difficult to interpret, but may suggest less engagement in VA care.

While having any type of GM/ID visit was similar during and prior to the COVID‐19 pandemic and by gender, we did identify differences in HIV care by gender. During both time periods, women were less likely than men to have in‐person GM/ID visits, to have VL measured and to be covered by ARVs. This finding is consistent with a previous study of the HIV care continuum using US VA data [21].

AUDIT‐C and smoking/tobacco use screenings were administered to a substantially lower percentage of PWH during the COVID‐19 pandemic. Before the pandemic, clinical staff usually administered the screenings during in‐person visits. It is likely that during the pandemic, in‐person visits were more focused on urgent issues and, therefore, routine screening questionnaires may not have been administered as frequently (this could be similarly true for virtual visits in 2019). Early in the pandemic, nurses triaged and screened only in‐person visits, leaving providers to add these screenings to their workflow. These findings indicate that providers were able to adapt to administering the screenings during virtual visits without the same level of support staff. This is likely emblematic of a rapid uptake of virtual care modalities to replace in‐person visits in 2020, as opposed to ad hoc, urgent or interim virtual assessments in 2019, which would be less likely to include routine preventive healthcare. Alcohol and tobacco use screening was done less frequently in 2020 during both in‐person and virtual visits and this represents an important area for improvement.

Later in the pandemic, nurses started calling, triaging and screening patients before virtual visits. However, the timing and process for this change likely varied by site, may not be reflected in these data, and warrants follow‐up research. Although providers took on the additional workload of administering the alcohol/tobacco screenings during virtual visits, this may not be an efficient or effective use of time in the long term. Integrating the use of support staff during virtual visits may be a way to improve screening. While ARV coverage was maintained during the pandemic, the lower frequency of substance use screening may have deleterious implications for other preventive care measures and to overall health outcomes in the coming months–years.

This study has some limitations. We excluded 2689 people who died or tested positive for SARS‐CoV‐2 during the study timeframe, which could have excluded PWH who were particularly vulnerable. Compared to those excluded, the 27,674 included were slightly more likely to be women (3.7% vs. 2.9%), younger (mean = 58.7 vs. 61.1 years) and less likely to be of Black race (45% vs. 51%). Most of the PWH are men so results for women may not generalize to non‐VA PWH populations. However, we believe these concerns are addressed in the results by race/ethnicity and gender (Table 2). The similar percentage with detectable HIV‐1 RNA VL between 2019 and 2020 should be interpreted with caution because there is a higher percentage without measured VL in 2020 and missing VL may be associated with lower ARV use. Additional follow‐up time is needed to determine whether those without a VL measurement in 2020 are of similar health status as those without a VL measurement in 2019. However, it is reassuring that ARV coverage is similar before and during the pandemic and suggests that aspects of the differentiated service delivery during the COVID‐19 pandemic may be worth continuing post‐pandemic. Of note, ARV coverage was lower for March 2019 than for any other month, and we surmise this is because we included those identified in VACS up to 1 March 2019 and there are likely some PWH who were new to HIV care up to this date.

## 5 CONCLUSIONS

With the emergence of the COVID‐19 pandemic, the US VA substantially increased the use of virtual (mostly telephone) visits and longer refills (mostly by mail) for ARVs, maintaining a high percentage of patients with suppressed VL among those with VL measured. Despite a lower level of in‐person services for PWH during the pandemic, access to ARVs was not disrupted. More observation time is needed to determine whether the health of PWH, measured by VL suppression, CD4 cell count, comorbidity diagnoses and other long‐term outcomes, was impacted by the differentiated service delivery and to evaluate whether a long‐term shift to increased use of virtual healthcare could be beneficial, particularly for those in rural areas or with transportation barriers. Findings could have long‐term implications for more efficient HIV care in general, perhaps involving longer prescription fills, greater use of mail in prescription services, fewer in‐person visits and less frequent VL tests.

Future research should evaluate whether newly diagnosed PWH had more challenges achieving VL suppression during the COVID‐19 pandemic. In the short term, it is reassuring that the level of GM/ID visits and ARV use remained consistent and that healthcare during this period of the US COVID‐19 pandemic was similar for PWH of Black, White, Hispanic and other race/ethnicities. However, a concerning finding is that ARV use and VL testing were lower for women than men both before and during the pandemic. Further studies are needed to evaluate the assessment of and treatment for HIV and substance use during the COVID‐19 pandemic.

## COMPETING INTERESTS

VCM received research grants from Gilead Sciences and ViiV, and served as an advisory board member for Eli Lilly and Company and Novartis. Otherwise, the authors declare that they have no competing interests.

## AUTHORS' CONTRIBUTIONS

KAM, ACJ, KMA, JPT, JTKJr, CTR, VCM, EH, FK and RY contributed to the conception and design MS, LSP and FK were responsible for data acquisition. KAM, ACJ, KMA, JPT, JTKJr, EH, CT R, JE and LS Park contributed to the analysis and interpretation. All authors contributed to the first draft or to critical edits and revisions of a subsequent draft. All authors have approved the final version of the manuscript and have agreed to be accountable to all aspects of the work.

## FUNDING

This study was funded by the National Institute on Alcohol Abuse and Alcoholism (grants U24‐AA020794, U01‐AA020790 and U10‐AA013566‐completed), Emory University Center for AIDS Research (grant P30AI050409) and the United States Department of Veterans Affairs Health Services Research & Development (grant C19 21‐287). The funders of this study had no role in study design, data collection, analysis, interpretation and presentation or in the decision to submit the manuscript for publication. Views presented in the manuscript are those of the authors and do not reflect those of the Department of Veterans Affairs, or the US Government.

## References

[1] Fultz SL , Skanderson M , Mole LA , Gandhi N , Bryant K , Crystal S , et al. Development and verification of a 'virtual' cohort using the National VA Health Information System. Med Care. 2006;44(8):S25–30. 16849965 10.1097/01.mlr.0000223670.00890.74

[2] Heyworth L , Kirsh S , Zulman D , Ferguson JM , Kizer KW . Expanding access through virtual care: the VA's early experience with Covid‐19. NEJM Catal Innov Care Deliv. 2020;1(4).

[3] Ohl ME , Richardson K , Rodriguez‐Barradas MC , Bedimo R , Marconi V , Morano JP , et al. Impact of availability of telehealth programs on documented HIV viral suppression: a cluster‐randomized program evaluation in the Veterans Health Administration. Open Forum Infect Dis. 2019;6(6):ofz206. 31211155 10.1093/ofid/ofz206 PMC6559276

[4] Wilkinson L , Grimsrud A . The time is now: expedited HIV differentiated service delivery during the COVID‐19 pandemic. J Int AIDS Soc. 2020;23(5):e25503. 32378345 10.1002/jia2.25503 PMC7203569

[5] Collins LF , Colasanti JA , Nguyen ML , Moran CA , Lahiri CD , Marconi VC , et al. The COVID‐19 pandemic as a catalyst for differentiated care models to end the HIV epidemic in the United States: applying lessons from high‐burden settings. AIDS. 2021;35(2):337–41. 33165032 10.1097/QAD.0000000000002746 PMC7775326

[6] Reddy A , Gunnink E , Deeds SA , Hagan SL , Heyworth L , Mattras TF , et al. A rapid mobilization of “virtual” primary care services in response to COVID‐19 at Veterans Health Administration. Healthc (Amst). 2020;8(4):100464 32992109 10.1016/j.hjdsi.2020.100464 PMC7434426

[7] Connolly SL , Stolzmann KL , Heyworth L , Weaver KR , Bauer MS , Miller CJ . Rapid increase in telemental health within the Department of Veterans Affairs during the COVID‐19 pandemic. Telemed J E Health. 2020;27:454–8. 10.1089/tmj.2020.0233 32926664

[8] Rosen CS , Morland LA , Glassman LH , Marx BP , Weaver K , Smith CA , et al. Virtual mental health care in the Veterans Health Administration's immediate response to coronavirus disease‐19. Am Psychol. 2021;76(1):26–38. 33119331 10.1037/amp0000751

[9] Baum A , Kaboli PJ , Schwartz MD . Reduced in‐person and increased telehealth outpatient visits during the COVID‐19 pandemic. Ann Intern Med. 2021;174(1):129–31. 32776780 10.7326/M20-3026 PMC7429994

[10] Spelman JF , Brienza R , Walsh RF , Drost P , Schwartz AR , Kravetz JD , et al. A model for rapid transition to virtual care, VA Connecticut primary care response to COVID‐19. J Gen Intern Med. 2020;35(10):3073–6. 32705471 10.1007/s11606-020-06041-4 PMC7377306

[11] Department of Veterans Affairs VHA Telehealth Services [Intranet] Available from: http://vaww.telehealth.va.gov/technology/covid19‐tech.asp. Accessed 2 Jul 2021.

[12] Ferguson JM , Jacobs J , Yefimova M , Greene L , Heyworth L , Zulman DM . Virtual care expansion in the Veterans Health Administration during the COVID‐19 pandemic: clinical services and patient characteristics associated with utilization. J Am Med Inform Assoc. 2021;28(3):453–62. 33125032 10.1093/jamia/ocaa284 PMC7665538

[13] Department of Veterans Affairs . HIV/hepatitis C QUERI strategic plan. Washington, DC: US Department of Veterans Affairs; 2010.

[14] Thompson MA , Mugavero MJ , Amico KR , Cargill VA , Chang LW , Gross R , et al. Guidelines for improving entry into and retention in care and antiretroviral adherence for persons with HIV: evidence‐based recommendations from an International Association of Physicians in AIDS Care panel. Ann Intern Med. 2012;156(11):817–33. 22393036 10.7326/0003-4819-156-11-201206050-00419 PMC4044043

[15] Williams EC , McGinnis KA , Edelman EJ , Matson TE , Gordon AJ , Marshall BDL , et al. Level of alcohol use associated with HIV care continuum targets in a national U.S. sample of persons living with HIV receiving healthcare. AIDS Behav. 2019;23(1):140–51. 29995206 10.1007/s10461-018-2210-6 PMC6344313

[16] Ridgway JP , Schmitt J , Friedman E , Taylor M , Devlin S , McNulty M , et al. HIV care continuum and COVID‐19 outcomes among people living with HIV during the COVID‐19 pandemic, Chicago, IL. AIDS Behav. 2020;24(10):2770–72. 32382823 10.1007/s10461-020-02905-2 PMC7203502

[17] Hitch AE , Gause NK , Brown JL . Substance use screening in HIV care settings: a review and critique of the literature. Curr HIV/AIDS Rep. 2019;16(1):7–16. 30747409 10.1007/s11904-019-00434-9

[18] Updated recommendations on service delivery for the treatment and care of people living with HIV. Geneva: World Health Organization; 2021. 33999550

[19] Ohl ME , Richardson K , Rodriguez‐Barradas MC , Bedimo R , Marconi V , Morano JP , et al. Impact of availability of telehealth programs on documented HIV viral suppression: a cluster‐randomized program evaluation in the Veterans Health Administration. Open Forum Infect Dis. 2019;10;6:ofz206. 10.1093/ofid/ofz206 PMC6559276 31211155

[20] McGinnis KA , Brandt CA , Skanderson M , Justice AC , Shahrir S , Butt AA , et al. Validating smoking data from the Veteran's Affairs Health Factors dataset, an electronic data source. Nicotine Tob Res. 2011;13(12):1233–9. 21911825 10.1093/ntr/ntr206 PMC3223583

[21] Matson TE , McGinnis KA , Rubinsky AD , Frost MC , Czarnogorski M , Bryant KJ , et al. Gender and alcohol use: influences on HIV care continuum in a national cohort of patients with HIV. AIDS. 2018;32(15):2247–53. 30005010 10.1097/QAD.0000000000001946 PMC6136970

[22] Abdel‐Rahman O . Patient‐related barriers to some virtual healthcare services among cancer patients in the USA: a population‐based study. J Comp Eff Res. 2021;10(2):119–26. 33448874 10.2217/cer-2020-0187

[23] US Department of Veterans Affairs 172VA10P2: VHA Corporate Data Warehouse – VA. 79 FR 4377. Office of the Federal Register, National Archives and Records Administration; 2020.

[24] Bradley KA , DeBenedetti AF , Volk RJ , Williams EC , Frank D , Kivlahan DR . AUDIT‐C as a brief screen for alcohol misuse in primary care. Alcohol Clin Exp Res. 2007;31(7):1208–17. 17451397 10.1111/j.1530-0277.2007.00403.x

[25] McGinnis KA , Skanderson M , Edelman EJ , Gordon AJ , Korthuis PT , Oldfield B , et al. Impact of behavioral and medication treatment for alcohol use disorder on changes in HIV‐related outcomes among patients with HIV: a longitudinal analysis. Drug Alcohol Depend. 2020;217:108272. 32971391 10.1016/j.drugalcdep.2020.108272 PMC7757793

[26] Akbar LS , Warshany LK , Kalathil LA , Autrey LK . Assessment of consolidated mail outpatient pharmacy utilization in the Indian Health Service. Fed Pract. 2020;37(7):325–30. 32908337 PMC7473714

[27] US Department of Veterans Affairs . Pharmacy Benefits Management Services. VA Mail Order Pharmacy. Available from: https://www.pbm.va.gov/PBM/CMOP/VA_Mail_Order_Pharmacy.asp. Accessed 16 Mar 2021.

[28] Price‐Haywood EG , Burton J , Fort D , Seoane L . Hospitalization and mortality among black patients and white patients with COVID‐19. N Engl J Med. 2020;382(26):2534–43. 32459916 10.1056/NEJMsa2011686 PMC7269015

[29] Rentsch CT , Kidwai‐Khan F , Tate JP , Park LS , King JT Jr , Skanderson M , et al. Patterns of COVID‐19 testing and mortality by race and ethnicity among United States veterans: a nationwide cohort study. PLoS Med. 2020;17(9):e1003379. 32960880 10.1371/journal.pmed.1003379 PMC7508372

[30] Rodriguez‐Diaz CE , Guilamo‐Ramos V , Mena L , Hall E , Honermann B , Crowley JS , et al. Risk for COVID‐19 infection and death among Latinos in the United States: examining heterogeneity in transmission dynamics. Ann Epidemiol. 2020;52:46–53.e2. 32711053 10.1016/j.annepidem.2020.07.007 PMC7375962

[31] McGinnis KA , Fine MJ , Sharma RK , Skanderson M , Wagner JH , Rodriguez‐Barradas MC , et al. Understanding racial disparities in HIV using data from the veterans aging cohort 3‐site study and VA administrative data. Am J Public Health. 2003;93(10):1728–33. 14534229 10.2105/ajph.93.10.1728 PMC1448041

